# CARM1 arginine methyltransferase as a therapeutic target for cancer

**DOI:** 10.1016/j.jbc.2023.105124

**Published:** 2023-08-01

**Authors:** Margarida Santos, Jee Won Hwang, Mark T. Bedford

**Affiliations:** Department of Epigenetics & Molecular Carcinogenesis, The University of Texas MD Anderson Cancer Center, Houston, Texas, USA

**Keywords:** CARM1, Tudor domain, epigenetics, cancer, small molecule inhibitors, cancer therapeutics

## Abstract

Coactivator-associated arginine methyltransferase 1 (CARM1) is an arginine methyltransferase that posttranslationally modifies proteins that regulate multiple levels of RNA production and processing. Its substrates include histones, transcription factors, coregulators of transcription, and splicing factors. CARM1 is overexpressed in many different cancer types, and often promotes transcription factor programs that are co-opted as drivers of the transformed cell state, a process known as transcription factor addiction. Targeting these oncogenic transcription factor pathways is difficult but could be addressed by removing the activity of the key coactivators on which they rely. CARM1 is ubiquitously expressed, and its KO is less detrimental in embryonic development than deletion of the arginine methyltransferases protein arginine methyltransferase 1 and protein arginine methyltransferase 5, suggesting that therapeutic targeting of CARM1 may be well tolerated. Here, we will summarize the normal *in vivo* functions of CARM1 that have been gleaned from mouse studies, expand on the transcriptional pathways that are regulated by CARM1, and finally highlight recent studies that have identified oncogenic properties of CARM1 in different biological settings. This review is meant to kindle an interest in the development of human drug therapies targeting CARM1, as there are currently no CARM1 inhibitors available for use in clinical trials.

Coactivator-associated arginine methyltransferase 1 (CARM1), also known as PRMT4, is a member of the protein arginine methyltransferase (PRMT) family. This family consists of nine PRMT proteins (PRMT1-9) (1), as well as a 10th more distant relative, NDUFAF7, whose function is restricted to the mitochondria (2). Based on their catalytic activity, PRMTs are split into two major types: type I PRMTs, like CARM1, that deposit asymmetric dimethylarginine (ADMA) marks, and type II PRMTs that deposit symmetric dimethylarginine marks.

Most PRMTs methylate their substrates within glycine/arginine-rich (GAR) motifs, which are composed of RGRG or RGGRGG repeats (3). In contrast, CARM1 methylates a rather loosely defined proline/glycine/methionine motif (4) that usually harbors a proline residue close to the arginine residue targeted for methylation (5). With few exceptions, CARM1 methylation sites are generally not recognized by other PRMTs (4, 6); thus, inhibition or knock out (KO) of CARM1 is not well-compensated for by other PRMTs due to a lack of functional redundancy.

Arginine methylation does not alter the charge of the arginine residue, but rather, generates a docking site for effector proteins that “read” this posttranslational modification (7). These effectors generally harbor Tudor domains, which selectively read either symmetric dimethylarginine or ADMA marks. The primary effector for CARM1-deposited ADMA marks is TDRD3 (8). TDRD3 is tightly complexed with topoisomerase IIIB (TOP3B) (9, 10) and recruits TOP3B to CARM1-methylated substrates (10). TOP3B can target both DNA and RNA by resolving negative supercoiled regions of DNA and RNA knots and concatenates (11). The majority of CARM1 substrates are involved in either the regulation of transcription or RNA processing, and the ability of its substrates to recruit the TDRD3/TOP3B complex is often key for executing the biological effects of CARM1. Importantly, there are other effectors for CARM1 methylation motifs that do not harbor Tudor domains, and these include TRIM29, PAFc, and FOXO3a (7).

PRMTs are often dysregulated in different cancer settings (12), and there is a strong interest in developing therapeutic strategies to target them. Here, we will focus on why CARM1 should be regarded as a viable therapeutic target in several different cancer types. We will start by highlighting what is known about the normal roles of CARM1 with respect to *in vivo* mouse models, CARM1 substrates that are implicated in transformation and cancer and CARM1-related preclinical studies. We hope to provide the reader with a broad understanding of CARM1 function in both normal physiological and cancer settings, which will also set the stage for how to prioritize those cancer types that would likely respond best to CARM1 targeting, what biomarkers can be used to stratify these treatments, and what we might expect as off-target or side-effects of prolonged CARM1 inhibitor (CARM1i) treatment in cancer patients. Importantly, small molecule inhibitors which are very specific for CARM1 have been developed but are not yet suitable for use in clinical trials. Hopefully, we can spur the scientific community into embracing CARM1 as a therapeutic target and developing drug-like compounds to inhibit CARM1 for use in the clinic.

## CARM1 mouse models

**Figure 1:**
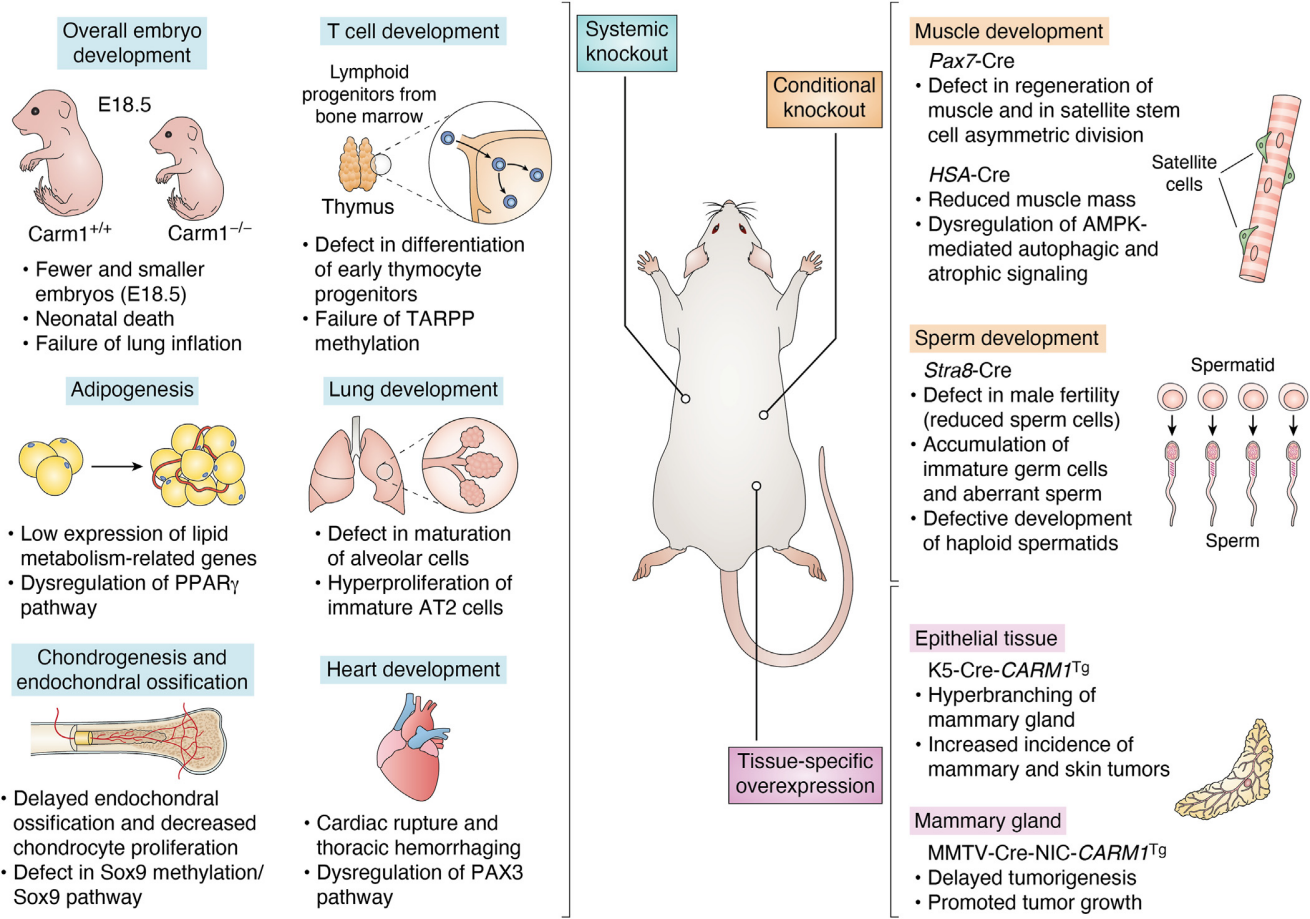
**An overview of various differentiation processes that Carm1 regulates in mice, summarizing the primary phenotypes highlighted and discussed in the review**. This information derives from several genetically engineered mouse models, including full systemic KOs of CARM1, conditional CARM1 KOs and a transgenic model of CARM1 conditional overexpression. AMPK, AMP-activated protein kinase; AT2, alveolar epithelial type II; CARM1, coactivator-associated arginine methyltransferase 1; HAS, human alpha-skeletal actin; K5, Keratin 5 promoter; MMTV, mouse mammary tumor virus promoter; NIC, neu-IRES-Cre; Pax7, paired box 7; Pax3, paired box 3; PPARγ, peroxisome proliferator-activated receptor gamma; Sox9, SRY-box transcription factor 9; TARPP, thymocyte cyclic AMP-regulated phosphoprotein.

The study of genetically engineered mouse models (GEMMs) that manipulate *Carm1* has provided great insight into the “normal” function of this enzyme. These studies offer a well-controlled background to understanding the biological roles of CARM1 and may help predict the impact of CARM1 inhibition in a human disease setting (Fig. 1).

## CARM1 KO models

### Full systemic CARM1 KO

The KO of *Carm1* with a neomycin insertion into the *Carm1* locus in mice was published in 2003 (13). The KO construct allowed for the deletion of neomycin by crossing with a flippase expressing mouse, which reactivates the locus and leaving behind two floxed exons that can be removed to create *Carm1* conditional KOs for the studies described below. *Carm1*^*−/−*^ embryos are present in normal Mendelian ratios at mouse developmental stage E15.5. However, by E18.5 the number of *Carm1*^*−/−*^ embryos is lower than expected, and they are smaller than their WT counterparts. *Carm1*^*−/−*^ pups die shortly after birth and these neonates fail to inflate their lungs. Poly(A)-binding protein 1, one of the first identified substrates for CARM1 (5), is not methylated in mouse embryonic fibroblasts isolated from *Carm1*^*−/−*^ embryos, suggesting a lack of redundancy with other PRMTs. Transcriptome analysis showed that CARM1 plays a role as a coactivator in transcriptional regulation *in vivo*, as first demonstrated *in vitro* by Mike Stallcup’s group (14). A key takeaway from this work is that the loss of *Carm1* is fairly well tolerated during embryonic development, in contrast to both *Prmt1* (15) and *Prmt5* (16) KOs, which display very early embryonic lethal phenotypes. Thus, CARM1i will likely have a much wider therapeutic window than either PRMT1 or PRMT5 inhibitors, as PRMT1 and PRMT5 have many important housekeeping functions.

### Follow-up studies using the systemic CARM1 KO

Although *Carm1*^*−/−*^ embryos die perinatally, it is possible to glean information regarding CARM1 function from these embryos. (1) CARM1 is important for proper T cell development. Based on the finding that CARM1 can methylate thymocyte cAMP-regulated phosphoprotein, a protein expressed in immature thymocytes, T cell development was analyzed in E18.5 *Carm1*^*−/−*^ embryos (17). Researchers observed a partial developmental arrest of the earliest thymocyte progenitor subset (CD44+ CD25-) as well as dramatically reduced cellularity. Subsequently, fetal liver competitive reconstitution assays revealed that *Carm1*^*−/−*^ cells are defective in multiple hematopoietic lineages (18). (2) CARM1 functions in adipogenesis as a coactivator for the transcription factor PPARγ, which was revealed by transcriptome analysis of *Carm1* KO embryos at E18.5 (19). (3) CARM1 regulates chondrogenesis and endochondral ossification through methylation of the transcription factor SRY-box transcription factor 9 (SOX9) (20). *Carm1*^*−/−*^ embryos display delayed ossification and accelerated cartilage development. Mechanistically, this phenotype is driven through a disruption of SOX9/β-catenin interaction and the downregulation of cyclin D1 expression. (4) CARM1 is important for lung development and lung cell renewal. *Carm1*^*−/−*^ pups die shortly after birth because they fail to take their first breath (13, 21). A close analysis of the lungs of these newborn mice revealed a lack of alveolar cell maturation, and hyperproliferation of immature AT2 cells (21). Furthermore, *Carm1*^*+/−*^ heterozygotes are susceptible to chronic obstructive pulmonary disease when challenged with cigarette smoke (22), implicating CARM1 in airway epithelial regeneration and repair. Thus, CARM1 plays a role in lung development and function. (5) CARM1 is critical for normal heart development, as revealed through an N-ethyl-N-nitrosourea mutagenesis screen (23), with many *Carm1*^*−/−*^ mid-gestation embryos displaying cardiac rupture and hemorrhaging in the thorax. This phenotype may be due to the dysregulation of the PAX3 transcription factor pathway, especially given that PAX3 can be methylated by CARM1 (24), and *Pax3* mouse KO embryos have cardiac defects that are very similar to *Carm1*^*−/−*^ embryos (25).

### CARM1 conditional KO mouse models

CARM1 binds to and methylates the transcription factor PAX7, which plays a key role in myogenesis (24). Therefore, a conditional KO of CARM1 in the skeletal muscle was developed to examine the role of CARM1 in muscle development. *Carm1*^*flox*^ mice were crossed with *Pax7-Cre* mice to create the conditional KO. In these mice, CARM1 is needed for satellite stem cell asymmetric division and entry into myogenesis. Functionally, methylation of PAX7 by CARM1 acts as a molecular switch for elevating the expression of the myogenic determination gene *Myf5*. More recently, a human alpha-skeletal actin promoter-driven *Cre* was used to knock out *Carm1* in skeletal muscle (26). These KOs display reduced muscle mass and altered AMP-activated protein kinase activity, which in turn affects downstream autophagic signaling. Transcriptomic analysis showed that *Carm1* regulates muscle atrophy (27). Interestingly, morpholino-mediated KO of CARM1 in zebrafish also results in defective muscle development: CARM1 is required for fast fiber formation by regulating myogenin expression (28). *Carm1* has also been genetically ablated from mouse male germ cells using a *Stra-Cre* mouse line which revealed that although CARM1 is not required for spermatocyte development, it is required for late-stage haploid spermatid development, where it counteracts the positive transcriptional activity of the p300/ACT/CREMτ axis (29).

### The CARM1 enzyme-dead model

Many enzymes possess scaffolding functions in addition to their catalytic function (30). When considering the effects of small molecule drugs inhibitors of CARM1, it is important to separate these potential functions. For example, CARM1 interacts with and activates poly (ADP-ribose) polymerase 1 (PARP1) in an enzyme-independent manner during replication fork PARylation (31), and scRNA-seq analysis of MDA-MB231 breast cancer (BC) cells, following either *Carm1* KO or CARM1i treatment, shows a distinct CARM1-KO population (not found in the inhibitor-treated cells) on a t-distributed stochastic neighbor embedding plot (32), suggesting that CARM1 loss may have a more profound effect on MDA-MB231 cell differentiation than CARM1i treatment. Nonetheless, an enzyme-dead *Carm1* knock-in mouse model indicates that the majority of CARM1 function derives from its methylation activity, and therefore, CARM1-specific small molecule inhibitors will eliminate the majority of CARM1 functions (33). For example, the enzyme-dead knock-in model recapitulates the KO with respect to (a) reduced mutant embryo size; (b) block in T-cell development; (c) loss of thyroid hormone responsive protein expression; (d) the impaired interaction between coactivator of 150 kDa and survival motor neuron protein; and (e) attenuated estrogen receptor (ER) coactivator activity.

### The CARM1 over-expression mouse model

CARM1 is not highly mutated in cancers, but it is often overexpressed. A GEMM for *Carm1* overexpression was developed using a strong ubiquitous promoter and a floxed STOP cassette (29). Tissue-specific Cre recombinase expression excises the STOP cassette, which then allows for ectopic *Carm1* expression. Using this approach, *Carm1* has been overexpressed in keratin 5-expressing epithelial tissues (K5-Cre) and in mouse mammary glands using mouse mammary tumor virus-Cre together with an oncogenic driver, mutant ERBB2/Neu (Neu-IRES-Cre [NIC] mice). Nulliparous K5-Carm1 mice display hyperbranching of the mammary glands, but only very late in life (∼18 months). In addition, these mice display an increased incidence of both spontaneous mammary tumors and skin tumors with median onset >20 months, suggesting that CARM1 overexpression predisposes epithelial tissues to transformation. However, the long latency of tumor onset in K5-*Carm1* females suggests that overexpression of CARM1 alone is not a major cancer-initiating event but may instead cooperate with an oncogenic insult. This concept was investigated using NIC mice. Interestingly, NIC-CARM1 mice develop tumors more slowly (∼2 weeks) than NIC mice, but the *Carm1* overexpressing tumors grow much larger. Thus, elevated *Carm1* levels initially delay tumorigenesis, but then promote tumor growth once the tumor initiates in the mouse mammary gland.

### CARM1 double KO mouse models

Although CARM1 has a methylation motif distinct from other PRMT motifs, some CARM1 substrates can be methylated by other PRMTs. For example, both CARM1 and PRMT6 can methylate histone H3 R17 (H3R17) *in vitro*. Given *Prmt6* KO mice are viable and normal in size (34), a *Prmt6 Carm1* double KO was created (6). The double KO embryos are noticeably smaller than their *Carm1*^*−/−*^ littermates and the H3R17me2a mark is lost, providing *in vivo* evidence for a degree of redundancy between these two PRMTs. No other PRMT-*Carm1* double KO mice have been generated to date.

A take-home message from the various mouse models described above and summarized in Figure 1, is that *Carm1* aids in driving cell differentiation and identity in conjunction with key transcription factors and other transcriptional coactivators.

## Transcriptional pathways regulated by CARM1

There are at least 1400 transcription factor (TF) genes in the human genome (∼6% of protein coding genes), and nearly half of these display tissue-specific expression patterns (35). In cancer, TF programs are often coopted as drivers of the transformed state and cancer cells can become dependent on these aberrant transcriptional programs, a condition referred to as TF addiction (36). This type of addiction occurs in both solid and liquid tumors (37, 38), but oncogenic TFs have proven very difficult to target with drugs (39). Such oncogenic TFs are often associated with the emergence of super-enhancers (40). CARM1, like the bromodomain-containing 4 protein (BRD4), is integral for the establishment and activity of enhancers (41, 42). Thus, inhibiting CARM1 may impede the development of tumors that rely on super enhancer –associated TFs. Here, we will highlight the different TF pathways with which CARM1 has been associated.

## Transcription factors that work with CARM1

### Nuclear receptors

The study describing the discovery of CARM1 by Mike Stallcup’s lab was one of the first to show that transcription factors can recruit methyltransferases as transcriptional coactivators, ultimately resulting in the modification of histone tails (14). This finding contributed to the histone code hypothesis proposal the following year (43), which states that transcription can be regulated by posttranslational modifications on histones. Using luciferase-reporter assays, they found that the p160 family of steroid receptor coactivators recruits CARM1 to nuclear hormone receptors (NR), including the ER, androgen receptor (AR), and thyroid hormone receptor to promote transcription. NRs have provided a great model system for studying the dynamics of CARM1 recruitment by TFs. Cryo-electron microscopy revealed that when CARM1 is recruited by the pre-existing ER/SRC3/p300 complex, it induces a structural change that promotes the activity p300, which then acetylates the K3K18 residue, and in turn promotes CARM1 activity on the adjacent H3R17 residue (44). CARM1 also directly methylates p300/CREB-binding protein (CBP) to regulate its histone acetyltransferase (HAT) activity. The ability of CARM1 to coactivate NRs may be clinically important for NR-driven cancers like BC and prostate cancer. Indeed, elevated levels of CARM1 are a prognostic factor for shorter survival in ER-positive BCs (45), although others have shown that CARM1 expression is higher in ER-positive low-grade tumors than in high-grade tumors, which may be linked to the differentiation status of these tumors (46). CARM1 is often expressed in two isoforms in breast tumors. The long isoform appears to have tumor suppressive function, whereas the short isoform (with exon 15 deletion) appears to have oncogenic function (47). The existence of these isoforms with different cancer-related functions complicates analyses that consider only total CARM1 protein levels. As discussed above, CARM1 overexpression mouse models show that CARM1 retards tumor initiation, but promotes progression once tumors are initiated (29). With regard to AR positive prostate cancer, fewer mechanism-based studies have been performed. However, correlative studies have found that CARM1 is elevated in early cancer progression, and further upregulated after androgen ablation therapy (48), suggesting that targeting CARM1 will have therapeutic value in both contexts.

### NFIB

A mass spectrometry-based CARM1 substrate screen identified the nuclear factor I (NFI) family of transcription factors (NFIA, NFIB, NFIC, and NFIX) as targets for CARM1 methylation (49). *Carm1* and *Nfib* KO mice phenocopy each other to some degree, with both KOs dying just after birth from lung tissue hyperplasia and respiratory defects (21, 50). NFIB is amplified in ∼10% of primary small cell lung cancers (SCLC) (51), and these tumors display increased chromatin accessibility (51, 52). Methylated NFIB recruits TRIM29, a transcriptional coactivator that helps promote the transcription of NFIB target genes. Follow-up studies focusing on the *Carm1/Nfib* axis reveal that the CARM1 methylation site on NFIB is critical for SCLC cell xenograft growth in mice. A SCLC GEMM with either a *Carm1* KO or a *Nfib* knock-in disrupting the methylation site, display at least a 50% increase in survival (from 200 days to over 300 days). In addition, studies using SCLC patient-derived xenograft models reveal that treatment with a CARM1i alone or in combination (CARM1i, etoposide, and cisplatin) has efficacy *in vivo*, as gauged by tumor volume after two months of treatment (49). These finding indicate that patients with NFIB-amplified SCLCs will likely respond to CARM1i, either as a monotherapy or in combination with etoposide/cisplatin.

### RUNX

There are two studies that provide evidence for cross-talk between CARM1 and RUNX1 (also called acute myeloid leukemia [AML] 1), a transcription factor that regulates genes with important functions in normal and malignant hematopoiesis (53). The first study, from the Nimer lab, was performed in the context of AML (54). They showed that RUNX1 is methylated by CARM1 on R223 leading to the recruitment of a repressor complex that inhibits expression of miR-223, a hematopoietic specific miRNA, with important functional outcomes in myeloid differentiation and leukemia. A second study, published by the Roeder and Chen Labs (55) showed that in the presence of AML1 (RUNX1)-ETO (AE) translocations, a unique stable transcription complex termed AML1-ETO-containing transcription factor complex (AETFC) is formed. AETFC preferentially binds to active enhancers and promotes AE-dependent gene activation. Gene activation involving AETFC also involves a direct interaction and chromatin recruitment of CARM1. CARM1 is required for the survival of cells bearing an AE translocation and is thus a good therapeutic target for this form of AML.

### PAX7

During myocyte differentiation, CARM1 interacts with PAX7 to specifically methylate multiple arginine residues in the PAX7 N terminus. Methylated PAX7 directly binds the C terminally cleaved forms of the H3K4 histone methyltransferases MLL1/2. This results in elevated expression of the PAX7 target gene *Myf5*, following asymmetric satellite stem cell divisions (24). A follow-up study showed that phosphorylation of CARM1 by mitogen-activated protein kinase 12 prevents CARM1 nuclear translocation in muscle stem cells (56). Further in dystrophin-deficient muscle stem cells undergoing asymmetric division the loss of dystrophin results in enhanced CARM1 phosphorylation, reduced CARM1 binding to PAX7, and decreased transcription of *Myf5* and other PAX7 target genes in progenitor cells. Thus, CARM1 plays a central role in regulating muscle stem cell function and myogenesis.

### SOX9

The transcription factor SOX9 regulates chondrogenesis (cartilage development) and ossification. In late development, *Carm1* KO embryos display delayed ossification (20). A molecular analysis of the cause of this phenotype revealed that SOX9 is a CARM1 interacting partner and substrate, which is needed for efficient cyclin D1 expression in chondrocytes.

## Transcriptional coregulators that work with CARM1

### CBP/p300

Shortly after the discovery of CARM1, it was found to methylate and regulate the activity of the secondary coactivators CBP and p300, two related HATs (57, 58). There is ordered cooperation between p300, PRMT1, and CARM1 for efficient transcription (59). The crosstalk between CARM1 and p300/CBP can lead to gene activation or repression in different contexts. In the context of the cyclic adenosine monophosphate signaling pathway, CARM1 functions as a corepressor, contrary to its coactivator function for nuclear hormones (58). CARM1 methylation sites on p300 (R580) and CBP (R601) reside within the KIX domain of these HATs, and when methylated, disrupt their interaction with CREB (58). During spermiogenesis, CARM1 also seems to function as a corepressor (60). CREMτ, a key testis-specific transcription factor, associates with p300 through its activator, ACT, and this interaction is negatively regulated by the methylation of p300 by CARM1. Additional CARM1-methylated sites on CBP, outside the KIX domain (R714, R742, and R768) (61), and in the C-terminal glucocorticoid receptor-interacting protein-1 binding regions of p300/CBP (62), play a role in glucocorticoid receptor-interacting protein 1--dependent transcriptional activation and in hormone-induced gene activation. A CARM1 methylation “code” was analyzed by *chromatin immunoprecipitation*-seq studies on CBP methylation sites revealing the recruitment of different methylated species to distinct estrogen-regulated genes (63). Importantly, this work also showed that methylation of CBP increased its acetyltransferase activity. In regard to the activating roles of CARM1 toward p300/CBP, the Santos Lab recently described a synthetic lethality between the dual loss of CBP/p300 HAT and CARM1 methyltransferase activity in the context of diffuse large B cell lymphoma (DLBCL) (64). CARM1 inhibition further reduces the HAT activity of CBP across the genome and downregulates CBP-target genes in DLBCL cells. Thus, there is potential for targeting CBP/p300-altered cancers with CARM1i or a combination of CARM1 and CBP/p300 inhibitors in specific cancers in which residual CBP/p300 expression is required for survival, as in DLBCL.

### β-Catenin

Wnt signaling results in both the stabilization of β-catenin and its translocation into the nucleus, where it functions as a coactivator of T-cell factor/lymphoid enhancer factor transcription factors and NR, like AR. CARM1 and β-catenin have synergistic effects that increase the transcription of AR and T-cell factor 4 in transcriptional reporter assays (65). CARM1 directly interacts with β-Catenin (66), and because there is crosstalk between AR and β-Catenin in castration-resistant prostate cancer, these cancers may be vulnerable to CARM1i (67).

### Mediator

The Mediator complex promotes transcription by facilitating the contact between enhancers and promoters through DNA looping, and high Mediator levels are associated with super-enhancers. CARM1 methylates components of the Mediator complex and is also a fairly stable part of this complex. A detailed analysis of Mediator in neural stem cells identified 75 interaction partners, including CARM1 and several CARM1 substrates (NCOA3, NFIB, and p300/CBP) (68). Within the Mediator complex, MED12 is the primary CARM1 target (42, 69), harboring at least three major methylation sites toward its C-terminal end. Methylated MED12 provides a docking motif for the Tudor domain-containing effector, TDRD3. The recruitment of TDRD3 positively modulates transcription of estrogen-regulated genes by stabilizing the interaction of MED12 with activating noncoding RNAs (42). The recruitment of CARM1 to MED12 is stabilized by Jumonji domain-containing protein 6 (70). The functions of MED12/CARM1 in transcriptional regulation occur through the Mediator kinase module. Recently, MED12/CARM1/p300 was found to function independently of the kinase module during immunoglobulin class switch recombination, during which it is critical for the establishment of the IgH super-enhancer (71), a process that likely also requires the TDRD3/TOP3B effector complex (10). MED12 is frequently mutated in human cancers, and these tumor suppressor functions might be augmented by its association with CARM1. Indeed, high levels of CARM1 and MED12 correlate with a better response to chemotherapy in BC patients (69). The methylation of MED12 appears to be required for the suppression of *p21/WAF1* expression, and upregulation of p21 has been associated with chemoresistance. Thus, combining CARM1i with chemotherapy may be deleterious in certain contexts, but high CARM1 protein levels could serve as a biomarker for a response to chemotherapeutic drugs.

### Switch/sucrose non-fermentable

There is strong evidence for reciprocal regulation between CARM1 and the switch/sucrose non-fermentable (SWI/SNF) chromatin remodeling complex, starting with the purification of the CARM1 protein complex years ago (72). This complex, termed NUMAC for nucleosomal methylation activator complex, harbors at least eight SWI/SNF components, including the ATPase Brahma protein-like 1 (BRG1/SMARCA4). Glycerol gradient fractionation of the CARM1 complex, isolated from MCF7 cells, revealed a solitary CARM1 complex copurifying with BRG1-associated factor 155 (BAF155) (BAF155/SMARCC1) and BRG1, suggesting that in MCF7 cells, CARM1 resides primarily in this complex. Importantly, recombinant CARM1 methylates the H3R17 site on free histone H3 (14), but is unable to modify this site in the context of a nucleosome. However, the NUMAC does methylate nucleosomal H3R17 (72). In addition, CARM1 stimulates the chromatin remodeling activity of SWI/SNF, which in the context of muscle cells, promotes myogenesis (73). Furthermore, BAF155 is a substrate for CARM1, and methylation of R1064 in BAF155 results in its redistribution to genes involved in the c-Myc pathway and promotes cancer metastasis in triple-negative breast cancer (TNBC) models (74). Methylation of BAF155 is also a prognostic marker for BC recurrence. Finally, in keeping with the common theme, methylated BAF155 colocalizes with BRD4 at super-enhancers, and CARM1i dramatically reduce the number of super-enhancers in TNBC cell lines (41). Combinatorial treatment of a mouse xenograft model of TNBC abrogates both tumor growth and metastasis. Although the SWI/SNF complex is a well-established tumor suppressor and it may seem counter-intuitive to inhibit CARM1 activity in this context, it is not. CARM1i have the potential for specifically targeting the oncogenic functions of SWI/SNF by both preventing the redistribution of this remodeling complex to genes involved in the c-Myc pathway and reducing the activity of tumor-associated super-enhancers.

### BRD4

Recently, BRD4 was identified as a CARM1 substrate (75). BRD4 is a transcriptional coactivator that is intricately associated with super enhancer–mediated transcription. CARM1, PRMT2 and BRD4 form a ternary complex. PRMT2, which harbors an SH3 domain, binds to the proline-rich region of BRD4 and recruits CARM1 to directly methylate BRD4 at a GAR motif that lies between its two bromodomains. Interestingly, GAR motifs are usually not CARM1 substrates, but the CARM1/PRMT2 complex clearly targets BRD4, suggesting that there may be something unique about this heterodimeric complex. BRD4 and the CARM1/PRMT2 complex coregulate a subset of genes, and the CARM1 small molecule inhibitor TP064 targets this same subset. Mutation of the methylation sites in BRD4 results in a reduced interaction with histone acetylated peptides and the dissociation of BRD4 from chromatin. Blocking BRD4 methylation attenuates cell proliferation *in vitro* and tumor growth *in vivo*. Furthermore, combinatorial treatment of BC cell lines with both CARM1 and BRD4 inhibitors is more effective than either single treatment, in both cell viability and colony forming assays. The ability of CARM1 to promote BRD4 function places it centrally in the regulation of super-enhancer–mediated transcription.

### PARP1

PARP1 has been implicated in the regulation of DNA repair, DNA replication, and transcription (76). It has long been known that PRMTs synergize with PARP1 in the regulation of NFκB-mediated gene expression (77). More recently, CARM1 was identified in an unbiased screen for proteins that associate with stalled replication forks in the process of restarting (31). CARM1 ablation experiments showed that it regulates fork restarting and promotes slow fork progression, but surprisingly, these processes do not require CARM1’s enzymatic activity. Instead, CARM1 binds directly to PARP1 to promote PARylation. It is thus possible that in the context of transcriptional regulation, CARM1 also promotes PARylation. It must be emphasized that CARM1i will not impact such regulation. However, the roles of PARP1 and CARM1 converge in other ways. For example, DNA double-strand break repair in mammalian cells occurs by homologous recombination (HR) and nonhomologous end-joining (NHEJ) and HR-incompetent cells are sensitive to PARP inhibitors. HR-competent cells can be forced to preferentially use the NHEJ machinery by overexpressing shieldin or one of its subunits, REV7, thus making the cells vulnerable to PARP inhibitors (78). It turns out that REV7 (MAD2L2) is transcriptionally repressed by enhancer of zeste 2 (EZH2) and activated by CARM1 (79). When cells are treated with EZH2 inhibitors, REV7 protein levels are dramatically increased, but only when the cells also express high levels of CARM1 to drive this switch. This provides both a mechanism (EZH2 inhibition) for a shift from HR to NHEJ, and a biomarker (high CARM1 levels) for conditions that permit this shift to make HR-competent cells vulnerable to PARP inhibitors. Thus, the functions of CARM1 and PARP1 may intersect in three distinct ways: first, CARM1 and PARP1 may work together to regulate gene expression; second, at restarting replication forks, CARM1 binds to PARP1 increasing its PARylation ability; and third, high CARM1 levels serve as a biomarker for vulnerability to PARP1 inhibitors in the presence of EZH2 inhibition. This indicates that CARM1 inhibitors might work to restore normal gene expression; they will not work to reduce CARM1-enhanced PARylation; and they will prove detrimental when CARM1 levels are used as a marker of vulnerability of cancer cells to EZH2 inhibition.

## The development of GlaxoSmithKline’s and Takeda’s CARM1i

The finding of the field discussed above strongly suggest that CARM1i will have therapeutic value, and cell-active chemical probes inhibiting CARM1 have already been developed both in pharmaceutical and academic settings. GlaxoSmithKline developed EZM2302 in collaboration with EpiZyme (80), and Takeda developed TP-064 together with the Structural Genomics Consortium in Toronto (81). Minkui Luo’s group at Memorial Sloan Kettering developed SKI-73, which is a chemical probe with prodrug properties. All three CARM1i have distinct molecular scaffolds. Their modes of interaction with CARM1 are also distinct, EZM2302 competes for the peptide-binding pocket and is stabilized by S-adenosyl-L-homocysteine, TP-064 also binds the peptide substrate-binding pocket and is SAM-noncompetitive, and SKI-73 engages the SAM-binding site and is cofactor-competitive. EZM2302 and TP-064 display antitumor activity against multiple myeloma (MM) (80, 81), AML (82), BC (32), and diffuse large B-cell lymphoma (64) in preclinical settings.

**Figure 2:**
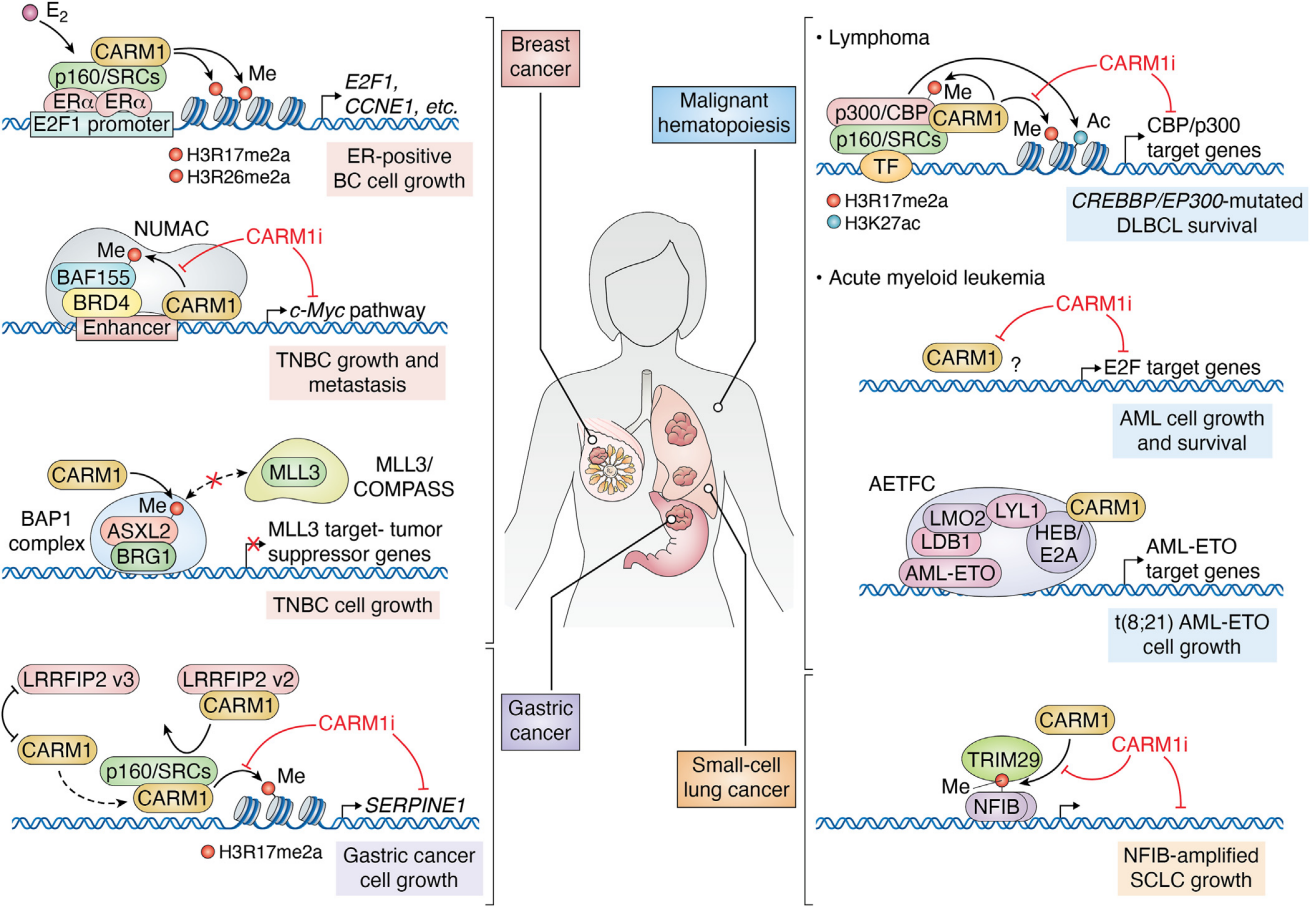
**An overview of the different human cancers in which CARM1 plays an oncogenic role and may be vulnerable to CARM1 inhibitor (CARM1i) treatment**. This figure summarizes the mechanisms by which CARM1 relays its transcription-promoting activities. AETFC, AML1-ETO-containing transcription factor complex; AML, acute myeloid leukemia; ASXL2, ASXL transcriptional regulator 2; BAF155, BRG1-associated factor 155; BAP1, BRCA1 associated protein 1; BRD4, bromodomain-containing protein 4; BRG1, Brahma-related gene 1; CBP, CREB-binding protein; CCNE1, cyclin E1 gene; CARM1, coactivator-associated arginine methyltransferase 1; COMPASS, complex proteins associated with Set1; DLBCL, diffuse large B cell lymphoma; DLBCL, diffuse large B cell lymphoma; ERα, estrogen receptors alpha; E2, estradiol; E2A, transcription factor E2-alpha; E2F1, E2F Transcription Factor 1; HEB, HeLa E-box-binding protein; LDB1, LIM domain-binding protein 1; LMO2, LIM domain only protein 2; LRRFIP2, LRR Binding FLII Interacting Protein 2; LYL1, lymphoblastic leukemia-derived sequence 1; MLL3, mixed-lineage leukemia protein 3; NFIB, nuclear factor I B; NUMAC, nucleosomal methylation activator complex; p160/SRCs, steroid receptor coactivators; SCLC, small-cell lung cancer; SERPINE1, serpin family E member 1; TF, transcription factor; TNBC, triple-negative breast cancer; TRIM29, tripartite motif containing 29.

Although the first of these CARM1i was described over 5 years ago, none have progressed past the chemical probe stage. To date, no CARM1i compound has received “Investigational New Drug” approval, and therefore no clinical trials can be initiated in this field. Thus, although preclinical studies strongly support the targeting of CARM1 in different cancer settings (Fig. 2), no clinical trials can be performed at this stage, which is impeding translational studies in the field.

## Evaluation of the prospects of targeting CARM1 in different cancer settings

### CARM1i in normal and malignant hematopoiesis

Early observations showing a role for Carm1 in hematopoiesis were made in *Carm1*-null and CARM1 enzyme-dead mice (17, 18, 33). Animals lacking either *Carm1* or its enzymatic activity presented with impaired fetal thymopoiesis (17, 33). Competitive reconstitution assays using *Carm1*-null fetal liver cells revealed a need for CARM1 for normal hematopoiesis. Further, impaired differentiation of *Carm1*-null fetal liver cells in an otherwise CARM1-intact host showed that CARM1 is required cell autonomously in fetal hematopoietic cells (18). Later studies performed in CD34+ human cord blood cells (54) revealed that overexpression of CARM1 blocks the myeloid differentiation of human stem/progenitor cells (HSPCs), and its knockdown induces their myeloid differentiation. CARM1 represses the expression of miR-223 in HSPCs *via* the methylation of RUNX1, a transcription factor that regulates genes with important functions in normal and malignant hematopoiesis (53). The same study analyzed gene expression data from AML patients and reported that *CARM1* levels were significantly upregulated in the AML samples than controls (54). Furthermore, depletion of CARM1 resulted in differentiation of myeloid leukemia cells *in vitro* and their decreased proliferation *in vivo*. This was the first study suggesting that CARM1 inhibition holds potential as a novel therapy for AML.

In 2018, two studies reported the first small molecule compounds (EZM2302 and TP-064) that potently and selectively inhibited CARM1, while also exhibiting antiproliferative effects on hematopoietic cells both *in vitro* and *in vivo* (80, 81). EZM2302 was tested on a diverse set of 36 hematopoietic cancer cell lines, and antiproliferative effects were observed with absolute IC_50_ values ranging from 0.015 to >10 μM (80), with the most potent antiproliferative effects seen in MM cell lines, with day 14 IC_50_ values of less than 100 nM. Oral dosing of EZM2302 also demonstrated dose-dependent *in vivo* CARM1 inhibition and antitumor activity in an MM xenograft model. Similarly, TP-064, is a potent, selective, and cell-active chemical probe of human CARM1 and its cocrystal structure with CARM1 has been described (81). TP-064 treatment inhibited the proliferation of a subset of MM cell lines, which displayed cell cycle arrest in G1. The following year (in 2019), the CARM1 inhibitor compound 49 was described as exhibiting good antitumor efficacy in AML cell lines *in vitro* and following oral administration in a xenograft model (83).

The first in-depth study of *Carm1* in normal and malignant hematopoiesis using a conditional KO mouse model and showing that CARM1 may be an effective therapeutic strategy for AML, was published in 2018 (82). This study showed that loss of *Carm1* has little effect on normal hematopoiesis. However, KO of *Carm1* abrogates both the initiation and maintenance of AML harboring *MLL* translocations (*MLLr* AML). This effect was accompanied by impaired cell-cycle progression, myeloid differentiation, and apoptosis. Patient xenografts as well as mouse models of AML were sensitive to CARM1 inhibition (EPZ025654) *in vivo*, showing significantly reduced AML cell growth and improved survival. More recent biochemical and genetic evidence also implicates CARM1 in the t(8;21) AE subtype of AML, which represents the most common fusion protein in AML (55). In this subtype, AE-containing complexes are heterogeneous, and assembly of the larger AETFC transcription factor complex, which can either activate or inhibit downstream genes, requires LYL1, which also recruits CARM1 to the AETFC complex to facilitate gene activation by AETFC, ultimately resulting in AML cell survival.

The involvement of CARM1 in lymphoma has been less well documented. CARM1 protein levels were reported to be higher in Hodgkin lymphoma cell lines than human germinal center (GC) B cells, and high CARM1 levels were detected in Reed-Sternberg cells in Hodgkin patient samples (84). Further, inhibition of CARM1 activity using EZM2302 slows nonHodgkin DLBCL growth in both *in vitro* models and *in vivo* xenograft models and the level of sensitivity to EZM2302 is positively correlated with the CBP/EP300 mutation load (64). Likewise, a combination of CARM1 and CBP/p300 inhibitors had a strong synergistic effect for the treatment of DLBCLs that lack CBP/EP300 mutations. Mechanistically, CARM1 inhibition reduces the HAT activity of CBP across the genome and downregulates its target genes in DLBCL cells, resulting in synthetic lethality. Consequently, the mutational status of CBP/EP300 may be leveraged as a biomarker for predicting the efficacy of small-molecule inhibitors of CARM1 in DLBCL and other cancers. Overall, in contrast to its key function in AML (both MLL-r and AML-ETO) and DLBCL, CARM1 appears to play a modest role in normal HSPC differentiation and proliferation (82), which indicates a favorable therapeutic value for CARM1 inhibitors in hematological malignancies.

### Breast cancer

CARM1 has been extensively studied in BC, likely because it was initially discovered as an ER transcriptional coactivator. Cell profiling experiments have shown that CARM1 protein levels are higher in BC tissue than normal breast tissue and positively correlate with human epidermal growth factor receptor 2 status and hormone-negative BC (45, 85, 86). CARM1 is required for ER-dependent cell cycle progression in BC cells both by promoting the expression of E2 promoter binding factor 1 (*E2F1*) transcription factor and acting as a coactivator for E2F1, thus regulating the expression of E2F1-target genes, such as the gene encoding the G1/S-specific cyclin E1 (87, 88). A later study showed that overexpression of CARM1 reduced cell growth and colony formation, while increasing differentiation in ERα-positive MCF7 cells, but not in ERα-negative MDA-MB-231 cells, suggesting CARM1 may function in reprogramming ERα-regulated cellular processes (46). Mediator complex protein MED12 is methylated at R1862 and R1912 by CARM1 in BC cells, and mutation of these sites in cell lines leads to resistance to chemotherapy drugs. Importantly, the protein expression levels of CARM1 and MED12 are positively correlated, and their high expression also predicts better prognosis in human BCs after chemotherapy (69). Jumonji domain-containing protein 6 is necessary for both the stable interaction of MED12 with CARM1 (70) and for ERα-dependent BC cell growth and tumorigenesis, indicating that CARM1 inhibitors would be efficacious in the treatment of ERα-positive and endocrine-therapy-resistant BC (70).

CARM1 has been implicated in the regulation of several additional BC-related pathways that are ER-independent. The lysine-specific demethylase 1 (LSD1), is methylated by CARM1, which promotes the binding of the deubiquitinase USP7, resulting in stabilization of LSD1 protein (89). Using a large set of paired (normal/tumor) human BC tissues, a strong positive correlation between CARM1 and LSD1 was observed in the tumor samples. Stabilized LSD1 contributes to the transcriptional repression of E-cadherin and activation of vimentin, and consequently promotes invasion and metastasis of both ER+ and ER-BC cells. Furthermore, the arginine methylation level of LSD1 correlates with tumor grades in human malignant BC samples (89). CARM1 methylates pyruvate kinase M2, which reversibly shifts the balance of metabolism from oxidative phosphorylation to aerobic glycolysis in BC cells, creating a metabolic vulnerability that can be therapeutically exploited (90). Mechanistically, CARM1 methylation of pyruvate kinase M2 enhances its tetramerization and activity (91). BAF155 is also a CARM1 substrate in BC cells, and its methylation promotes tumor growth and metastasis, with methylated BAF155 displaying a chromatin association profile distinct from that of nonmethylated BAF155 (74). BAF155 methylation is also an independent prognostic marker for BC recurrence. A recent follow-up study revealed that arginine-methylation of BAF155 by CARM1 promotes TNBC metastasis in two independent ways: first, by activating super-enhancer-addicted oncogenes by recruiting BRD4; and second, by repressing the host immune response (41). The BAP1/MLL3 transcriptional activation complex subunit ASXL2 is methylated by CARM1, and this modification blocks binding to the methyltransferase MLL3 and impairs the expression of MLL3-regulated genes (92). Indeed, CARM1 loss or inhibition increases MLL3 chromatin recruitment and the activation of MLL3-dependent tumor suppressors. This transcriptional repressive function of CARM1 sheds light on the mechanisms involved in *MLL3* mutations seen in TNBC patients (93). Taken together, there is an abundance of evidence suggesting that CARM1 is a good target for both ER+ and ER-BC types.

### Ovarian cancer

Inhibition of EZH2 significantly suppresses the growth of CARM1-expressing, but not CARM1-nonexpressing, ovarian tumors (94). This selectivity correlates with apoptosis induction, and the reactivation of EZH2 target tumor suppressor genes in a CARM1-dependent manner. Mechanistically, CARM1 regulates the antagonism between SWI/SNF and EZH2 by methylating BAF155 (94). A related follow-up study showed that an EZH2 inhibitor sensitizes CARM1-high, but not CARM-low, HR-proficient epithelial ovarian cancer cells to PARP inhibitors (79). EZH2 inhibition upregulates *MAD2L2* in a CARM1-dependent manner to decrease DNA end resection, ultimately causing mitotic catastrophe in PARP inhibitor treated HR-proficient cells. A third study from the Zhang lab, reports that CARM1-expressing ovarian cancer cells are selectively sensitive to inhibition of the IRE1α/XBP1s pathway (95). In response to endoplasmic reticulum stress, the IRE1α RNase processes the mRNA encoding the transcription factor XBP1, leading to the translation of its spliced form, XBP1s. XBP1s translocates into the nucleus to promote the transcription of genes involved in protein folding as well as other targets to alleviate endoplasmic reticulum stress (96). In this context, CARM1 functions as a coactivator of XBP1s in determining the expression of the IRE1α/XBP1s pathway target genes. Inhibition of the IRE1α/XBP1s pathway was effective against experimental models of ovarian cancer in a CARM1-dependent manner (95). In all these ovarian cancer studies, CARM1 serves as a biomarker for cells amenable to EZH2 and/or PARP inhibition, rather than as a therapeutic target itself.

### Gastric cancer

High CARM1 expression has been correlated with poor prognosis in gastric cancer (97). CARM1-mediated transcriptional activation of the NOTCH2 signaling pathway is linked to NOTCH2 methylation in gastric cancer progression. Further, overexpression of a specific leucine-rich repeat (in FLII)-interacting protein 2 (LRRFIP2) splice variant that directly interacts with CARM1 and activates it, also contributes to the metastasis of gastric cancer cells (98). By inhibiting CARM1, the invasiveness of LRRFIP2-overexpressing gastric cancer cells can likely be repressed.

### Small-cell lung cancer

NFIB, a substrate for CARM1-mediated methylation, acts as an oncogene in SCLC (99) that both regulates chromatin accessibility and promotes SCLC metastasis (51, 52, 100, 101). High levels of NFIB are associated with human SCLC metastases and poor overall survival (52). Given that the CARM1 methylation site on NFIB is critical for SCLC cell growth in xenograft mouse models and that knockdown of CARM1, treatment with CARM1i, or loss of the NFIB methylation site leads to better survival in these models, targeting CARM1 provides a therapeutic avenue to reduce NFIB oncogenic activity in SCLC (49). Importantly, NFIB is also amplified in ER-negative BC and esophageal squamous cell carcinoma (102, 103, 104), and targeting CARM1 may attenuate its oncogenic roles in these cancers as well.

## Cancer immunotherapy

Broadly speaking, there are two types of immunotherapies, either active or passive, which include chimeric antigen receptor-T cell therapy or immune checkpoint inhibitors, respectively. CARM1 can promote checkpoint inhibition, as shown through being the top hit in a CRISPR screen designed to identify negative regulators that enhance T cell proliferation/survival within tumors using B16F10 melanoma cells (105). The same study also found that CARM1 inhibition enhances the antitumor functions of CD8 T cells and promotes the maintenance of tumor-infiltrating T cells that express memory markers. Thus, CARM1 loss provides a double whammy, both sensitizing tumors to T cell-mediated killing and preventing the depletion of the active T cell population. Indeed, B16F10 cells, which are resistant to monotherapy with checkpoint antibodies (CTLA4 and DP-1), become sensitive if CARM1 is knocked out or if xenograft model mice are treated with CARM1 inhibitors. This sensitizing phenotype is driven through MED12 (as the CARM1 substrate) and TDRD3 (as the methyl-effector) (105). Mechanistically, CARM1 is required to stabilize the interaction between MED12 and histone H3, and the loss of CARM1 reduces terminal differentiation gene signatures. Indirect evidence for a role of CARM1 inhibition in promoting chimeric antigen receptor-T cell therapy was also uncovered through a CRISPR screening approach (106). In this case, the screen was designed to identify genes that, when lost, effectively enhance T cell potency after editing and recovered MED12 (the major CARM1 substrate) as the top hit, along with its associated kinase regulator, Cyclin C. Although CARM1 was not identified in this screen, it is worth testing, considering its close link (as a positive regulator) with MED12, in many contexts. Thus, the use of CARM1 inhibitors to enhance cancer immunotherapies may be of great value in the future.

## Possible side effects of CARM1i treatment

Based on over two decades of study in various *Carm1* mouse models, it is clear that loss of CARM1 is fairly well-tolerated. This is not the case for CARM1-related enzymes like PRMT1 and PRMT5. Thus, CARM1 inhibitors will likely have a larger therapeutic window than PRMT1/5 inhibitors. Nonetheless, side effects of long-term CARM1 inhibitor exposure in humans are possible and may include negative effects on male fertility, loss of muscle mass, development of osteoporosis, degeneration of lung airways, and development of heart disease. Long-term treatments could also result in the possible impairment of humoral immunological responses, because CARM1 regulates CBP activity in GC-derived B cell lymphomas, and this likely happens during normal GC reactions as well. However, GCs are highly dynamic; therefore, this impairment is expected to be reversible following cessation of treatment. It should also be noted that despite the many roles of CARM1, remarkably, CARM1 inhibition does not dramatically impair hematopoiesis.

## Summary

CARM1 inhibition is expected to have a positive impact on the lives of patients afflicted with a large number of cancer types. Here, we have presented strong evidence from mouse and cell models, supporting the use of CARM1i in the context of cancer. A few key points have emerged: (1) CARM1 is tightly associated with enhancer activity, and it is key for the establishment of super-enhancers that are established in cancers that display TF addiction; (2) biomarkers like NFIB amplification, p300/CBP, and MLL3 mutations can serve as indicators for CARM1 inhibitor efficacy; (3) depending on the cancer, CARM1 inhibitors in combination with other inhibitors may be effective, including BRD4, EZH2, and PARP1 inhibition; (4) cancer immunotherapy will likely benefit from combinatorial treatment with CARM1 inhibitors; and (5) because we have already defined many of the normal biological roles of CARM1, we also have a framework for monitoring for possible side-effects of inhibitor treatment in patients.

## Conflict of interest

The authors declare the following financial interests/personal relationships which may be considered as potential competing interests. Mark T. Bedford is the co-founder of EpiCypher.
